# Return-To-Play Decision Making in Team Sports Athletes. A Quasi-Naturalistic Scenario Study

**DOI:** 10.3389/fpsyg.2020.01020

**Published:** 2020-06-03

**Authors:** Jochen Mayer, Stephanie Burgess, Ansgar Thiel

**Affiliations:** ^1^Institute of Sports Sciences, Faculty of Social Sciences, University of Göttingen, Göttingen, Germany; ^2^Institute of Occupational and Social Medicine, University Hospital Tübingen, Tübingen, Germany; ^3^Institute of Sports Science, Faculty of Economics and Social Sciences, University of Tübingen, Tübingen, Germany

**Keywords:** return-to-play, return-to-sport, culture of risk, playing hurt, active information search, risky decision making, team sports

## Abstract

Competitive athletes act within cultures of risk in sports and often decide to return to sport despite having acute health problems. The outcomes of such risky return-to-play decisions can not only negatively affect their future health, but may also limit their sports performance or even upset their career paths. Following risk-management-decision theory with its focus on active risk defusing, we developed a model for understanding the process of return-to-play decision making from an athlete’s perspective. Based on the method of active information search, a quasi-naturalistic return-to-play decision scenario was created in order to assess amateur team sport athletes’ decision-making strategies. The main goals were to identify different information acquisition patterns and to analyze the influence of varying sporting consequences on decision making. A total of 72 competitive team sport athletes (36 females, 36 males, *m* = 25.7 years of age, 3rd to 6th league level) from three disciplines (volleyball, basketball, and handball) participated in the experimental study. Facing the same medical scenario (a partial tear of the supraspinatus tendon), athletes show different approaches to return-to-play decision making. The main focus is on the potential sporting consequences of withdrawal from competition due to injury, with only a few players favoring well-informed decisions based on thorough risk analysis. The athletes who chose the medically risky alternative to play hurt mostly employed strategies of active risk defusing, which got activated when severe sporting consequences were perceived. Those who chose to withdraw from competition primarily referred to maximin heuristic, particularly when social pressure to play was reduced. The findings can be used to improve rehabilitation-related communication and shared return-to-play decision making in sports.

## Introduction

Decisions about whether to return to play or rest when having an acute health problem are typical of all kinds of sports. Such choices usually have to be made under time pressure induced by competitive schedules and refer to uncertainties about both sporting and medical consequences. They are further characterized by the need to justify them to significant others like coaches, managers, team members, or even the public. From the perspective of the injured or ill athlete, the decision’s potential consequences can not only negatively affect their future health, but also their sports performance and long term personal goals. For example, competing hurt can have the positive outcome of being part of a possibly winning team. Yet, choosing this alternative could result in the negative outcome of aggravating the injury. On the other hand, choosing to rest may be beneficial in terms of the healing process, but might lead to various sporting consequences such as losing one’s position in the team. Therefore, return-to-play decisions have to be considered as “risky decisions” (Huber, 2012), which are generally defined by at least one uncertain negative outcome in at least one of the alternatives. In the following, we will focus on the question of how athletes proceed when confronted with risky return-to-play decisions.

From a sports medicine perspective, return-to-play decisions are mostly discussed as clinicians’ decisions of when and how an athlete is allowed to return to sport after rehabilitation. This includes injury-specific recommendations for doctors which are almost exclusively derived from expert opinion and clinical experience (Huang et al., 2016). A common orientation for return-to-play decisions, irrespective of who has the decision-making authority, is the model of “Strategic Assessment of Risk and Risk Tolerance (StARRT)” (Creighton et al., 2010; Shrier et al., 2015). The key elements of this framework are the assessment of the health risk (step 1), the assessment of activity risk (step 2), and the assessment of risk tolerance (step 3). Particularly, step 3 includes the so-called decision modifiers which point out to the necessity of including sports network related expectations into deciding, including seasonal phase, the potential to mask the injury, or external pressures. The normative StARRT-model comprehensively includes available research, clinical evidence, and expert opinion. However, it does not intend to capture the process of actual decision making.

Team doctors, who define medical diagnoses, inform patients about treatment options, and set return-to-play schedules, act as mediators between the sport-related expectations and the culture of precaution of the medical system (Safai, 2003). Clinical practice and empirical research show that return-to-play decision authority varies to a relevant extent and stakeholder groups often have heterogeneous opinions on which criteria should be considered when deciding (Shultz et al., 2013; Shrier et al., 2014). At the same time, there is social pressure on team doctors in order to minimize lay-off times and clear athletes for competitions (Malcolm, 2006). Consequently, painkillers are often used and medical treatments are timed in accordance to the relevance of upcoming games and the seasonal stage (Murphy and Waddington, 2007; Roderick, 2006).

Another restriction of the StARRT-model is that it does not consider the perspective of how athletes proceed in return-to-play decision-making situations. The athlete’s perspective, however, is particularly relevant. If athletes are perceived as fully competent, they are supposed to make an informed decision (Dijkstra et al., 2017). Thus, the athlete’s final decision is crucial, particularly if no physician is present or consulted at all. Sociological research indicates that the athletes’ decisions are made within a “culture of risk” which normalizes and glorifies taking health risks associated with sports participation (Nixon, 1992, 1993). “Playing hurt” is therefore a common phenomenon across all kinds of sports disciplines, age groups, and performance levels (Roderick et al., 2000; Schubring and Thiel, 2014). Thus, when deciding, long-term health perspectives are often neglected even by adolescent athletes in favor of short-term success in sport (Schnell et al., 2014). In this regard, the athletes’ willingness to compete not only varies, but is also highly affected by the sports disciplines’ culture of risk and the individually perceived social pressure to perform (Mayer and Thiel, 2018; Mayer et al., 2018).

Although there is a broad discourse about judgment and decision making in sports (Bar-Eli et al., 2011; Raab et al., 2019), no systematic studies exist about how athletes actually proceed in return-to-play decision-making situations. To date, decision-making research addressing athletes’ choices particularly focuses on typical sports action situations such as passing, shooting, or stroking in order to explain differences between expert and novice decisions or with the intention to test general decision making theories using sports as a study field (Raab et al., 2019). Such sports action situations are considered as a perfect performance environment to study perception based expert decision making, including the analysis of athletes’ perception and anticipation, attention, memory, and decisions made (Araújo et al., 2019). Most of the researched action situations are not only characterized by time constraints and short-term choices, but also by direct sensorimotor interaction with dynamic environments (Raab and Helsen, 2017). Yet, return-to-play decision making differs from these typical sports action related (single) task problems: Firstly, visual perception, other players’ movements anticipation, and bodily reactions are only of subordinate importance. Secondly, there is the opportunity to gather additional information about whether to play (or not) from several actors (e.g., doctors, coaches, physiotherapists) or other knowledge sources. Thirdly, return-to-play decision making hypothetically can be based on heuristic decision making, including simple or fast and frugal heuristics (e.g., Bennis and Pachur, 2006; Raab, 2012; Raab and Gigerenzer, 2015). However, it may also be grounded in more rational choice oriented deciding with subjective expected utility considerations (e.g., Johnson, 2006). Moreover, the return-to-play decision-making situation is usually embedded within a sport organizational context, so pre-programmed decision-making routines and cultural constraints are supposed to affect decision making processes as well (Mayer and Thiel, 2018). Against this background, we were looking for a suitable theoretical and methodical approach to research athletes’ return-to-play decision making procedures. This approach should be as naturalistic as possible, allow to analyze active information gathering processes and permit the incorporation of sociological findings about the culture of risk and playing hurt in sports organizations. As we expect that the above mentioned decision modifiers are supposed to play a crucial role in deciding about return-to-sport and playing hurt, the approach should also be flexible enough to include the practice related recommendations from the sports medical perspective.

**FIGURE 1:**
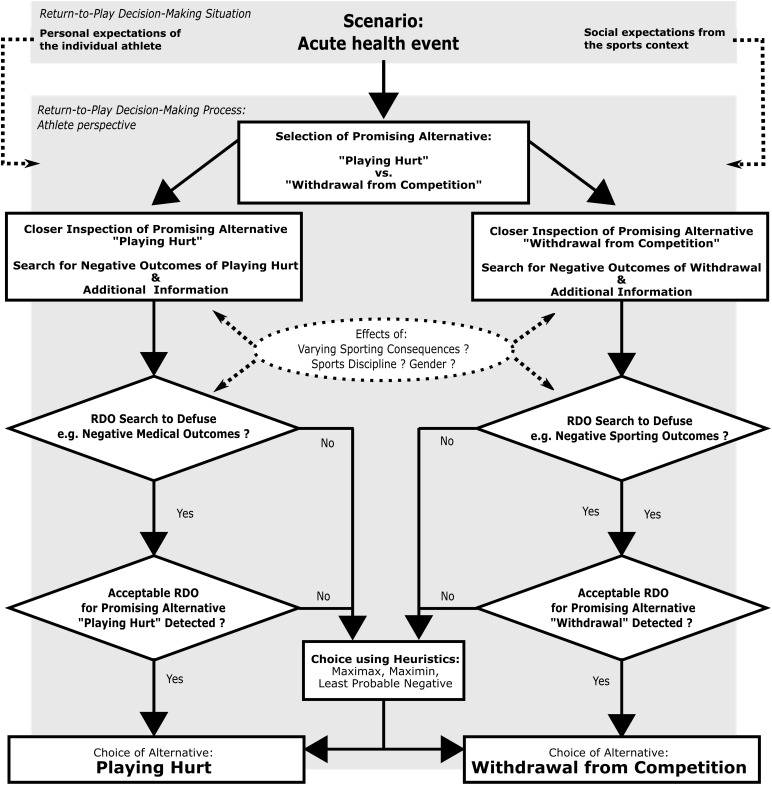
Process model for understanding return-to-play decision making from the athletes’ perspective following Risk Management Decision Theory (Huber, 2012).

A general framework that can help to better understand return-to-play decision-making strategies in consideration of decision modifiers is Huber’s (2012) “Risk Management Decision Theory (RMDT).” We transferred this broad risky decision theoretical approach to the sports context, adjusted its core ideas and developed a specific process model for understanding return-to-play decision making from an athlete’s perspective (see Figure 1). The process model captures the following theoretical considerations:

The decision-making process is initiated with the detection of a health event. According to the dynamic model of competing hurt (Mayer and Thiel, 2018), it is the specific nature of the underlying health event, which characterizes the initial decision-making situation. In this regard, the medical complexity of an acute, chronic, or episodic health problem predefines the level of uncertainty and riskiness of the situation in question. Another defining aspect of the decision-making situation is the characteristic sports context surrounding the individual athlete who is confronted with a health issue. Consequently, it does make a difference, whether the return-to-play decision is to be made toward the end of a longer injury induced rehabilitation process or if there is the need to immediately decide about further procedures in the case of an acute illness. Against this background, the following theoretical considerations will focus on those typical risky return-to-play decision scenarios which arise, whenever an acute health event is detected in competitive sports – irrespective of an athlete’s performance level, type of sport, age, or sex. This initial scenario functions as the starting point for the decision-making process.

According to RMDT, the process of individual decision making generally begins with the selection of the most promising alternative. In this respect, a decision maker constructs a rudimentary mental representation of the overall situation including all initially available alternatives. Following Huber (2012), this initial mental representation mainly consists of positive outcomes related to the alternatives. Concerning this, the alternative with the best outcome is usually selected as promising alternative. At this point, it is important to mention that the best outcome always refers to the subjective decision maker’s perspective. Transferred to return-to-play decision making, such a first representation likely consists of positive outcomes related to two alternatives, namely to either recover by withdrawing from sports or, on the other hand, to be able to participate in training or competition by playing hurt. This initial mental representation and the selection of the most promising alternative are supposed to be highly affected not only by an athlete’s personal preferences, but also by the formal and informal role expectations addressed by the sports environment.

In a next step, according to RMDT, the promising alternative is inspected in more detail. The decision maker searches for further information which leads to a more comprehensive mental representation. In this regard, decision makers appear to search in particular for information about negative outcomes (Huber, 2012). Transferred to athletes, negative medical consequences are likely to come into focus if the alternative to return-to-play hurt is closely inspected as the most promising one. In another case, an athlete favoring the alternative to withdraw from competition due to injury, might reflect about the negative consequences of this potential behavior, such as e.g., letting the team down. Against this background we hypothesize, that the severity of perceived sporting consequences of being absent affects the information search patterns and consequently influences athletes’ mental representation about their most promising option.

The subsequent step addresses the core concept of RMDT: It has to be decided whether or not to search for a so-called risk-defusing operator (Huber, 2012). A risk-defusing operator (RDO) is an action intended by the decision maker to be performed in addition to a risky, but at the same time attractive alternative (Bär and Huber, 2008). RDOs, as the core concepts of risky decision theory, are active means to decrease expected risks in the subjective perception of the decision maker. Different types of RDOs exist, such as those preventing a negative outcome or compensating negative consequences (Huber, 2007). Transferred to the sports context, it can be assumed that athletes – like people in other studies –do also include RDOs within the decision-making process while mostly excluding probability information. In return-to-sport decision making, a preventive RDO for the medically risky alternative (playing hurt) could be the use of additional protective gear, while a compensating one might be the availability of excellent health insurance. RDOs might also play a decisive role in compensating the negative sport related outcomes of the medically safe alternative (withdrawal from competition due to illness or injury). In this regard, the availability of an adequate substitute player could function as RDO as well.

Finally, as soon as an RDO has been explicitly detected or implicitly identified, it has to be decided whether it is acceptable in terms of cost-benefit ratio (Huber, 2012). In line with general RMD-theory, such explicit and implicit RDOs are supposed to play the decisive role within decision-making processes: If an acceptable RDO is found, then usually the associated risky alternative is chosen. Transferred to return-to-play-decisions, this means that an athlete very likely chooses to play hurt as soon as an appropriate way to subjectively defuse potential health risks is found.

If no acceptable RDO is identified or negative outcomes cannot be defused, general risk research shows, that decision makers usually select one of the available (promising) alternatives by using “heuristic decision making” (Gigerenzer and Gaissmaier, 2011). In this regard, heuristics are understood as “strategies reducing the cognitive effort necessary to make a decision” (Huber, 2012, p. 26). While RDOs are active ways to defuse the risk, the heuristics offered by classical decision theory are means of passive risk minimization. In addition to RDOs, Maximin heuristic is quite often used in risky decisions outside the sports context (Bär and Huber, 2008). Maximin compares the worst outcomes of alternatives and chooses the alternative with the least bad outcome. Other potential heuristics are Maximax, and Least Probable Negative. When using Maximax, the best outcome of each alternative is first identified, then the best outcomes are compared, and finally, the alternative with the best of the best outcomes is chosen. The Least Probable Negative heuristic is characterized by the identification of the worst outcome for each alternative and the choice of the alternative where the worst outcome has the lowest probability (Bär and Huber, 2008). Which RDOs and other heuristics are generally used, is supposed to depend on environmental and personals factors, such as the social situation or personality characteristics (Huber, 2012). Against this background, we assume the social context of sports with its particular social expectations (e.g., performance orientation, sports culture of risk) to fundamentally influence the decision-making processes. Specifically, we expect the perceived sporting consequences to affect the information search dependent activation of an acceptable RDO, which consequently leads to the choice of the most promising alternative. This mechanism might steer an athlete’s decision systematically toward the medically risky alternative of playing hurt if significant sporting consequences are perceived (e.g., losing one’s starting position in the team, missing an important match, or being stigmatized as weak or soft). Thus, we hypothesize that athletes choose the medically risky alternative more often if they perceive high sporting consequences – given the same nature of the underlying health event. On the other hand, given the same acute illness or injury, it can be assumed that those athletes who only perceive minor sporting consequences can easily prioritize health referring to heuristics such as Maximax. However, it is not yet clear, what kind of information is essential for athletes in making return-to-play decisions and if RDOs really do play a significant role for athletes as well. Moreover, it has to be empirically analyzed to what extent varying sporting consequences affect the risk evaluation process and RDO-inclusion. As athletes’ willingness to compete hurt is supposed to be related to the sport discipline (Mayer and Thiel, 2018), potential effects on the return-to-play decision-making process have to be considered as well. The same applies to potential gender effects, which too are the subject of controversy. Against this background, the developed process model (see Figure 1) summarizes the theoretical considerations made and functions as a comprehensive model for the empirical study of return-to-play decisions.

The main goal of the paper is to analyze athletes’ return-to-play decision-making strategies. In accordance with the presented theoretical framework we developed a quasi-naturalistic return-to-play decision-making scenario for amateur team sport athletes which allows to empirically address the following central research questions:

1. What kind of information search and acquisition patterns do athletes follow in a typical return-to-play decision scenario?
2. How do varying sporting consequences (SC) affect information acquisition strategies and RDO-inclusion within the return-to-play decision-making process?
3. What are the effects of RDO-inclusion and use of other heuristics on return-to-play decisions in relation to varying SC?

In addition to these central research questions we also assess whether sports discipline or gender is associated with information acquisition strategies, RDO-inclusion, and decisions made.

## Materials and Methods

### Return-to-Play Decision Making and the Method of Active Information Search

Based on the method of active information search (Huber et al., 2011a) we developed a quasi-naturalistic decision scenario for analyzing return-to-play decision making from an athlete’s perspective. Following Huber et al. (2011a), the basic idea of this method is the following: A short description of a complex risky decision situation including the alternatives is first given to the participants. To gather more information, the participants can then address questions to the experimenter, who responds to each question using standardized answers. The number and content of the questions are chosen by the participants, depending on their individual information needs for making a decision. Thus, participants dynamically construct and elaborate a mental representation of the decision-making situation (Huber, 2012). In this regard, the active interest of the participants in certain contents is taken as an indicator of the importance of specific kinds of information for deciding. Thus, the procedure allows to assess the subjective relevance of information about consequences, probabilities, or RDOs. It also helps to identify which informational categories are of initial importance, and to find out what kind of information leads to stopping the search process to decide. By manipulating the answers to specific questions, the effects of particular kinds of information on search processes and final decisions can be analyzed using experimental study designs. Additionally, decision makers can be told to justify their decisions afterward. Although RDOs usually play a prominent role in these personal justifications, indicators for other heuristics can also be traced back by content analysis (Bär and Huber, 2008). The method of active information search is mostly being used in fundamental risky decision making research (e.g., Huber and Huber, 2008; Huber et al., 2011b), but also in real-life genetic-counseling decisions (Shiloh et al., 2006), physicians’ diagnostic decisions (Kostopoulou et al., 2009), or in its thinking aloud version for the analysis of laypeople’s preferences for newly emerging climate engineering technology (Amelung and Funke, 2015). Against this background, the developed study design and procedure for analyzing athletes’ return-to-play decisions are presented in the following sections.

### Study Design and Procedure

The study was designed as a between-subjects experiment with performance oriented amateur team sport athletes as participants. The procedure started with proband enlightenment and obtaining informed consent, followed by a short questionnaire addressing participant background information and inclusion/exclusion criteria. The next step included an introduction to the scenario method. The general breeding turtles scenario (Huber and Kunz, 2007) was then used as a warming-up task. As the main task, the specifically developed “Supraspinatus return-to-play decision scenario” with its variation of sporting consequences (SC) was applied (detailed description below). The participating athletes were randomly assigned to one of the two sporting consequences groups (high sporting consequences vs. low sporting consequences). After deciding whether to compete or rest, the participants were asked to inform their coach about the choice and its justification by writing a (draft) email. The overall procedure took 25–50 min per athlete.

### Participants

A total of 72 competitive team sport athletes (36 females, 36 males, 25.7 years mean age, SD = 4.3) from different level contact sports (24 Volleyball, 24 Basketball, and 24 Handball) voluntarily participated. None of them had participated in a similar study before. The criteria for inclusion were: age 18–35, at least two training units per week, active participation in competitions within a team playing in the German league system (3rd to 6th league level), and no professional athletic experience. Exclusion criteria were: personal experiences with the injury described in the scenario, and current lay-off due to injury.

### The “Supraspinatus Return-to-Play Decision Scenario”

#### Scenario Development

The development of the quasi-naturalistic decision scenario followed a three-step process: First, an initial description for a typical return-to-play decision situation and a corresponding pool of answers to potential questions were created. For this, the basic procedure of the method of active information search (Huber et al., 2011a) was adjusted to the aims of our study and combined with scientific knowledge about return-to-play issues and practical knowledge taken from expert interviews with doctors and physiotherapists working in team sports settings. A central goal was to develop a scenario which allowed the inclusion of performance oriented amateur athletes from different team sports. The underlying rationale was to focus on a major and practically relevant target group which is often neglected. Second, pre-tests with professional handball players were conducted in order to create a comprehensive and highly realistic, but fictive scenario. Third, extensive pre-experiments with amateur team sport athletes from volleyball, handball, and basketball were performed to optimize the overall design and procedure, to enhance the wording and to improve the categorization of the answers.

The following considerations and experiences from pre-tests finally led to the “Supraspinatus return-to-play decision scenario.”

1. As the chosen medical condition highly affected the willingness to search for information, we decided to base our scenario on an ambiguous medical diagnosis, which basically allows an athlete to compete, but also comes with a relatively high risk of severe physical damage.
2. The participants are experts in the field as they basically know about general positive and negative outcomes of return-to-play decisions. To limit this expert status, we chose the medical condition “partial tear of the supraspinatus tendon,” which is relatively infrequent, but still typical for team sports with a significant upper body involvement. Thus, the precondition for a team sport to be included within this study was the shared characteristic, wherein a functioning shoulder joint is essential for the shooting movements and overall sports performance. In order to capture potential effects of sports discipline specific risk cultures, athletes from distinct team sports were recruited for the study, including a typical high contact sport (handball), a medium contact sport (basketball) and a non-contact sport (volleyball). Those athletes with personal experience about the scenario specific condition had to be excluded for the reason of limiting expert knowledge influence as well.
3. To minimize the influence of the subjects’ personal experiences from their own sports nets (e.g., personality of one’s coach or expertise of one’s team doctor) and to encourage information search, the amateur athletes were supposed to take over the fictive role of a professional handball player.
4. The exact wording of the introductory scenario description had to be very carefully adjusted in order to provoke information search and to avoid framing effects toward one of the two alternatives. Consequently, this description does not include information about possible consequences, RDOs, probabilities, or detailed situational information on sportive or medical issues, because the provision of those specific kinds of information can directly affect which alternative is taken. Extensive pre-tests showed, in particular, that including any information about SC within the introductory scenario description very likely resulted in immediate decision making without any further information requests. However, this approach included the risk that information about SC was not actively requested. A related challenge was the step-by-step preparation of predefined answers to all potential questions raised by the athlete during the information search process. In total, this process led to the creation of 95 different answers which finally covered the potential information requests. Each answer was printed on an index card. The answer cards were thematically organized in a cardfile box, allowing the experimenter to quickly present the right answer to each of the questions asked.
5. As subjects search more persistently for RDOs when they have to justify their decision afterward (Huber et al., 2009), the athletes were explicitly put under justification pressure by the directive to communicate the decision to the coach in writing.
6. To ensure that the athletes understood the central idea of the return-to-play decision scenario, which is the opportunity to request any information needed from the experimenter via the prepared answer cards, the inclusion of a general “warm up scenario” proved to be beneficial in addition to the standardized briefing about the procedure. In the following paragraphs, the resulting introductory scenario description and further information about data generation and statistical procedures are presented.

#### Introductory Description of “Supraspinatus Return-to-Play Decision Scenario”

The following short decision situation description was presented to the participants:

“Imagine the following situation: You are a professional handball player. For several days, you have had to deal with shoulder pain. During the training session on Thursday, the pain became so bad that you had to abandon the ongoing session. However, your team’s next game is scheduled for the following Saturday. Your coach immediately sends you to see the team doctor. The coach also tells you that he needs to be immediately informed about whether he can count on your participation in the upcoming competition. After an in-depth examination, the team doctor diagnoses a partial tear of the supraspinatus tendon. You must now decide between two alternatives. Which decision do you communicate to your coach? Alternative A: You decide to have a lay-off and withdraw from competition. Alternative B: You decide to participate in the game, despite the shoulder problem.”

The scenario description ended with the invitation to gather further information needed for deciding by simply asking any question(s) of personal relevance.

#### Standardized Answers

All questions asked by the participants during the decision process were categorized by the experimenter and answered non-verbally using the prepared index cards. This step was realized by matching the question to one of the 95 prepared answers. The answers on the index cards were assigned to a main category of questions in advance. Those main categories were developed in accordance to previous studies using the method of active information search (e.g., Bär and Huber, 2008) and adjusted to the case of return-to-play decisions:

##### Medical situation

All questions about the medical situation or medical background information. Example: What is the function of the supraspinatus tendon? How long is the healing process if I rest immediately?

##### Sporting situation

All questions about the general sporting background of the decision situation. Example: Which league is my team playing in?

##### Medical consequences

All questions referring to the medical consequences of an alternative. Examples: Will the injury get worse when I play?

##### Sporting consequences

All questions referring to sports network related consequences of an alternative. Examples: How important is the upcoming game? How essential am I for the team?

##### RDO

All questions about the existence of an RDO, including additional actions of prevention, compensation or worst-case plans. Example: Is there appropriate pain medication that would allow me to play?

##### Probabilities

All questions aiming at the uncertainty or probability of a consequence. A question did not necessarily have to contain the word “probability” to be classified as a probability question. Example: How likely is it that our team wins? How often does a tendon rupture happen?

##### Miscellaneous

Questions for which no standardized answers existed or requested information was already given. In this case, the subject got a card with the sentence: “There is no information regarding your question,” or “You already asked that question.”

#### Information Given About Varying Sporting Consequences

To identify the influence of social pressure to play hurt in competitive sports, we varied the sports related consequences of the action alternative “lay-off and withdrawal from competition.” The participants were randomly assigned to two conditions before the start of the procedure while ensuring same-sized subgroups with equal distributions of males and females, and participants from the three sport disciplines. The following independent variables were varied within participants: If participants assigned to the condition “low sporting consequences” asked a related question, the answers content implied only mild social expectations to play hurt. Participants assigned to the condition “high sporting consequences” were confronted with strong social pressure to compete. For example, the answers varied between “the upcoming game is a friendly match” vs. “the upcoming game is a final” or “the seasonal goal cannot be reached anymore” vs. “the seasonal goal is still within reach” or “the coach recommends a break” vs. “the coach expects you to play.”

#### Decision and Justification Texts

The information search process ended as soon as the participant athletes informed the examiner about their decision. The participants were then instructed to write an email to their coach informing about the decision and why this choice was made. The written document was then sealed in an envelope and put aside. The analysis of such written justification texts is a way to get insights into underlying decision heuristics and to clarify the role of RDOs for deciding (Huber et al., 2009). To analyze these texts, quantitative content analysis was used by applying the following general coding rules for decision heuristics as introduced by Bär and Huber (2008).

##### Maximax

The alternative with the best subjective outcome is chosen, whereas no probabilities or no negative consequences are mentioned. Example: I chose not to play because it is better to fully recover from an injury before practicing sports again.

##### Maximin

The alternative is chosen in which the negative outcome is least bad, whereas risks and negative consequences are mentioned, but no probability information is given. Example: For this game, it is not worth risking a more severe injury.

##### Least probable negative

The alternative is chosen where the negative outcome is least probable. Example: I rest because it is highly probable that severe long-term damage may occur if I play.

##### RDO assumption

Athlete assumes the existence of an RDO and chooses the alternative with the assumed RDO. Examples: I am only going to play for a few minutes, to support the team; or I will take painkillers to be ready to play.

##### Subjective expected utility (S)EU

This category is assigned when a participant refers to calculations involving outcome values and probabilities. This category does not presuppose that the decision maker does the calculations correctly or uses the appropriate probability. Example: The probability of rupturing the tendon is disproportional to the predicted 6 week lay-off time.

##### Not classifiable

If no decision code could be assigned. Example: I will play.

### Statistical Procedures

Pearson chi-squared tests and Fisher’s exact tests were calculated to assess the effects of varying sporting consequences, to find out about the associations between decisions made, information basis, heuristics, and RDO inclusion, and to control for gender and type of sport. Mann-Whitney U-Tests were performed when data was not normally distributed (based on Kolmogorov-Smirnov and Shapiro-Wilk Tests).

## Results

### Information Search

In total, 123 questions were asked by the participants. This results in a mean value of 1.71 (SD: 1.91) questions per athlete, with a minimum of zero and a maximum of 8 questions. Considering only athletes who actively searched for information, the mean value is 2.62 (SD: 1.79) questions.

#### Information Acquisition Patterns

Analyzing the information categories the athletes searched for, we could identify three distinct acquisition patterns:

1. No information category searched at all: Although the participants were well aware of the possibility of gathering information about the decision-making situation, 25 of them did not actively search for information. These 34.7% thus decided solely on the basis of incomprehensive scenario description and individually activated background knowledge.
2. Information search with focus on medical background and consequences only: The second search pattern was followed by 13 athletes (18.1%) and is characterized by a highly selective information search focusing solely on medical information. An average of 1.77 questions (SD: 1.01) were asked before deciding. Questions related to either medical situation (M: 0.92, SD: 0.49) or medical consequences (M: 0.85, SD: 1.07).
3. Information search including sports related information: A third search pattern was practiced by 34 athletes (47.2%). It is characterized by a search including sports related information categories and an average of 2.94 (SD: 1.92) questions asked. Within this group, two sub search patterns could be identified: The first one can be described as a comprehensive search strategy including medical and sporting, but also the occasional RDO and probability related information. The 20 athletes (27.7%) within this subgroup asked almost four questions on average (M. 3.8, SD: 1.93) with a focus on sporting consequences (M: 1.25, SD: 0.55), followed by medical background (M: 1.05, SD: 0.76), and medical consequences (M: 0.95, SD: 1.01) information. Only sporadically included were RDO related (M: 0.2, SD: 0.41) and probability-oriented questions (M: 0.1, SD: 0.31). The second sub search pattern, which includes 14 athletes (19.4%), is characterized by focusing solely on sporting information. Before deciding, these athletes asked 1.71 questions (SD: 1.07). However, most interest was also shown in questions about sporting consequences (M: 1.36, SD: 0.5), whereas questions about the sporting situation were only asked occasionally (M: 0.29, SD: 0.73). Within this group, only one athlete additionally requested RDO related information. No probability related question was asked.

While gender (χ^2^(2, *N* = 72) = 4.6, *p* > 0.05) is not significantly associated with a particular information acquisition strategy, an effect of the type of sport can be identified (Fisher’s exact test, *p* < 0.05). Apparently, most basketball players did not search for additional information at all (Basketball: 56% vs. Handball: 28% vs. Volleyball: 16 %). On the contrary, volleyball players most often showed search strategies including medical consequences and background information (Volleyball: 46.2% vs. Handball: 38.5% vs. Basketball: 15.4%) as well as sports related information (Volleyball: 41.2% vs. Handball: 35.3% vs. Basketball: 23.5%).

#### Relevance of Information Search Categories

To assess the overall relevance of RDOs and other information categories within the search process, we analyzed the questions asked for each category (see Table 1). Those athletes actively searching for information are mostly interested in the sporting consequences of having a break: Overall, 70.2% requested such kind of outcome related information. Neither sports discipline (χ^2^(2, *N* = 33) = 2.35, *p* > 0.05) nor gender (χ^2^(1, *N* = 33) = 0.503, *p* > 0.05) is significantly associated with a search for SC information. Further analysis reveals that those who search for SC-information are mostly interested in the relevance of the game (26 out of 44 questions asked), followed by questions about the player’s position within the team (10 out of 44), and the specific advice of the team doctor (4 out of 44).

**TABLE 1:** Relevance of information search categories.

Information search group (*N* = 47)	Overall relevance of categories info search		First alternative related question		Last alternative related question	
	*N*	%	*N*	%	*N*	%
Sporting consequences	33	70.2	15	31.9	27	57.4
Medical situation	26	55.3	27	57.4	7	14.9
Medical consequences	17	36.2	3	6.4	10	21.3
Sportive situation	6	12.8	2	4.3	2	4.3
RDO	5	10.6	–	–	1	2.1
Probability	2	4.3	–	–	–	–

Of the second highest importance is general information about the medical situation, as it is requested by 55.3%. Within this category, most interest is taken in features of the injured structure (23 out of 33 questions asked) and its function (7 out of 33). Neither types of sports (χ^2^(2, *N* = 26) = 1.21, *p* > 0.05) nor gender (χ^2^(1, *N* = 26) = 1.16, *p* > 0.05) is associated with this kind of information request.

The third highest interest is taken in medical consequences (36.2%). Again, neither types of sports (fisher’s exact test, n.s.) nor gender (χ2(1, *N* = 17) = 0.34, *p* > 0.05) is associated with this kind of information request. Information demands about potential physical damage of playing (15 out of 30) and expected lay-off times (8 out of 30) were most common. Only of minor interest is information about the general sportive situation (12.8%) and possible RDOs (10.6%). Questions addressing probability were extremely rare (4.3%).

To determine which information categories have the highest initial and final subjective importance for the decision process, the first and the last questions of those athletes actively searching were examined. The analysis of the very first question indicates which one of the alternatives is considered initially as the most promising one. For almost two thirds of the athletes, playing hurt is the most promising alternative, as medical background and is of initial importance for 57.4 and 6.4% respectively. The alternative to rest is initially the most promising one for the remaining athletes demanding SC (31.9%) and sporting situation specific information (4.3%). RDO or probability information are not of initial importance. Neither gender nor the type of sport is associated with the first alternative related question category (Fisher’s exact tests, n.s.).

The last alternative related question searched for is supposed to point out which type of information is crucial to stop the search process and to finally make a choice. In this regard, SC information leads most athletes (57.4%, *N* = 27) to stop the search process to make a final decision. However, for 21.3% (*N* = 10), it is information about medical consequences which leads to a termination of the search process. Thus, for 78.7% of all athletes, it is potential outcome related information that is of central importance. However, medical background information (14.9%, *N* = 7), sporting situation (4.3%, *N* = 2), and RDO information (2.1%, *N* = 1) also lead to stopping the search in some cases. Probability information is not important with regard to stopping the search. Again, neither gender nor the type of sport is significantly associated with the last alternative related question category (Fisher’s exact tests, n.s.).

A closer examination of these very few individual search strategies including RDO questions reveals that this kind of information is always requested toward the end of a longer search process and only if negative medical consequences were detected first. Focusing solely on the cases asking for probability information shows that this kind of information is of interest late within the search process (not before the sixth question) and is followed by further assessment of information about consequences afterward.

#### Sub-Group Analysis: Associations Between Sporting Consequences and Information Search

In the following paragraph, we focus on the effects of high or low SC on information search variables, including the search for RDOs. Athletes receiving high SC information do search for RDOs more often (4 out of 18, 22.2%) than the ones confronted with low SC (1 out of 15, 6.6%). However, this difference is not significant (Fisher’s exact test, n.s.). Those athletes who identified high SC do search for more different kinds of information (*m* = 2.28, SD 1.41) than the ones who face only minor SC (*m* = 1.87, SD 0.83) and ask more questions (*m* = 3.39, SD 2.06) than the other group (*m* = 2.27, SD 1.58). However, these differences are not significant either (Mann-Whitney U-Test, ns.).

In both conditions, the most relevant category before the final decision is SC, as 27 out of 33 athletes (81.8%) make their choice after getting this kind of information. However, there are no significant differences between the two groups regarding the category of the last information searched for (Fisher’s exact test, n.s.).

### Association Between Return-to-Play Decisions, Information Base, and Perceived Sporting Consequences

Overall, 43% of all athletes chose the medically risky alternative to play despite the shoulder injury, while the remaining 57% decided to cancel the upcoming competition in order to rest. There is a significant association between the information basis (no info search at all, no SC search, high or low SC), and the final decision made (χ^2^(3, *N* = 72) = 20.75, *p* < 0.01), with Cramer’s V measure of effect size indicating a large effect (0.537). Those who actively searched for SC information and who got confronted with low SC all chose the medically safe alternative to rest (100%). The ones confronted with high SC information chose to play hurt in 77.8% of the cases while only 22.2% decided to rest. Subgroup analyses including only the 33 athletes requesting SC information consequently reveal a significant difference between the two conditions (χ^2^(1, *N* = 33) = 20.26, *p* < 0.01) with Cramer’s V measure of effect size (0.784) indicating a considerably large effect. The search group not requesting any SC information tends to choose the medically non-risky alternative more often (64.3%), while those athletes not requesting any information at all choose to rest more often (52%) than to compete hurt (48%). Overall, no associations were found between gender and choice (χ^2^(1, *N* = 72) = 0.57, *p* > 0.05) or type of sports and choice (χ^2^(2, *N* = 72) = 2.15, *p* > 0.05).

### Effects of RDO-Inclusion and Other Heuristics on Final Return-to-Play Decisions

In the following section, the choices are further analyzed in terms of underlying decision heuristics with regard particularly to the influence of high and low SC. In a first step, the results of the quantitative content analysis of justification texts are presented.

#### Decision Making Heuristics Based on Justification Texts – Quantitative Content Analysis

Quantitative content analysis reveals that athletes refer to distinct justification strategies when explaining their decisions (see Table 2). Most justification strategies are associated with three decision making heuristics: Maximin, RDO, and Maximax. Information related to heuristics such as Least Probable Negative or Subjective Expected Utility (SEU) is only rarely identified within the texts. The most common justification strategies refer to Maximin-heuristics (47.2%, *N* = 34). To justify recovery, 44% of the athletes mention the risk of severe medical consequences (e.g., aggravation of injury, long-term injury, or sports invalidity) as too high compared to the potential negative outcomes of not being part of the team. In contrast, they rarely refer to Maximin in order to justify the choice to play. Only 2.8% of the players mention that the negative health consequences are considered at least as bad as the negative sporting ones.

**TABLE 2:** Justification texts and assigned heuristics by final choice.

Heuristic	Choice	Content (n)	Choice n (%)	Heuristic n (%)
Maximin	Play	I‘ll play although it hurts / even if this is bad for my health (2)	2 (2.8%)	34 (47.2%)
	Rest	For this game, it is not worth risking a more severe injury (10); I do not want to take the risk of a long-term injury/sports invalidity (10); If I play, the risk of a long lay-off due to a more severe injury/rupturing the tendon is too high (8); If I rest, I am out for just a few weeks which is better than having to rest months with a tendon rupture (3); High Risk (1)	32 (44.4%)	
RDO	Play	To support the team, I am going… to play for just a few minutes/…at less than 100%/…stop playing when pain increases during the match (10); To support the team, I postpone the lay-off until the next match is over (4); I will get my shoulder tape bandaged before the match (4); Take painkillers to be ready to support team (3); The doctor does not categorically exclude the option to play (2); Excellent treatment options after a potential tendon-rupture leads me to play (1)	24 (33.3%)	27 (37.5 %)
	Rest	An adequate substitute is available so I can rest to be back soon (3)	3 (4.2%)	
Maximax	Play	I want to support the team/I don‘t want to abandon the team (4); The upcoming match is very important and the team needs me (1)	5 (6.9%)	8 (11.1%)
	Rest	It is reasonable to fully recover from an injury before practicing sports again (3)	3 (4.2%)	
Least probable negative	Rest	It is highly probable that severe long-term damage occurs if I play, and that I am also not 100% fit to play (1); There is a 50/50 percent chance that the tendon ruptures, so the risk of a 6-month recovery time is too high (1)	2 (2.8%)	2 (2.8%)
(S)EU	Rest	The probability of tendon rupture is disproportional to the predicted lay-off time of 6 weeks (1)	1 (1.4%)	1 (1.4%)

While only 7% (*N* = 5) of the athletes actively searched for RDOs, analysis reveals that 37.5% (*N* = 27) mentioned an RDO in the justification texts. RDOs are mainly included to justify the alternative to play hurt (33%, *N* = 24). In this regard, the most common RDO is to support the team by either playing for just a few minutes, at less than 100% capacity or by stopping playing when the pain increases during the match (*N* = 10). Other common RDOs are to postpone the recovery (*N* = 4), getting one’s shoulder taped (*N* = 4), and taking pain killers (*N* = 3). Yet, a few athletes who chose to rest referred to an RDO too (*N* = 3). In these cases, having an adequate substitute ready to play is seen as an appropriate action to reduce the sporting risks related to one’s recovery (4.2%, *N* = 3).

Justification strategies associated with Maximax heuristics are used by 11.1% of all the players. These strategies are applied to justify both choices. For some athletes, it is simply necessary to support the team (6.9%), while for others it is more reasonable to recover from an injury before practicing their sport again (4.2%). Only three athletes (3.2%) mention probabilities in their justifications at all. Even where probabilities are included, they are only used to justify the decision to rest. Two athletes refer to the chances of possible physical damage, which is typical for the least probable negative heuristic. Indications for risk calculations involving probabilities and outcome values as characteristic for SEU can only be found in one of the three texts including probabilities at all.

#### Association Between the Final Decision and Underlying Decision Heuristics

In a next step, associations between underlying decision heuristics (including RDO inclusion) and return-to-play decisions are analyzed. By doing so, the already identified association between high SC and the choice to play hurt is further evaluated to find out whether the detection of an acceptable RDO is associated with this medically risky alternative (see Table 3). Overall, there is a significant association between the inclusion of an RDO to defuse the medical consequences of competing and the decision made (χ^2^(1, *N* = 72) = 47.61, *p* < 0.01). Such RDOs were included in 77.7% of all justification texts of those athletes who chose to play, while no such information was referred to when taking the alternative to rest. The Cramer’s V measure of effect size was large (0.813).

**TABLE 3:** Decision heuristic used in relation to information search type and sporting consequences condition.

	Information search and information type condition (n)				
Decision heuristic by alternative (based on justification texts)	Info search: high sporting consequences	Info search: low sporting consequences	Info search: no sporting consequences	No info search	Overall n (%)
**Decision: play hurt**	**14**	**–**	**5**	**12**	**31 (43.1%)**
RDO (medical risks)	13	–	3	8	24
Maximax	1	–	1	3	5
Maximin	–	–	1	1	2
Least probable negative	–	–	–	–	–
SEU	–	–	–	–	–
**Decision: Rest**	**4**	**15**	**9**	**13**	**41 (56.9%)**
RDO (sporting risks)	–	2	1	–	3
Maximin	2	13	6	11	32
Maximax	–	–	1	2	3
Least probable negative	2	–	–	–	2
SEU	–	–	1	–	1
**Overall n (%)**	**18 (25.00%)**	**15 (20.83%)**	**14 (19.45%)**	**25 (34.72%)**	**72**

Subgroup analysis of decision making heuristics including only those who asked for sporting consequences (*N* = 33) reveals the following: The identification of an acceptable RDO predicts the choice to play hurt in 92.86% of the cases when high social risks of a lay-off are included, while the Maximax heuristic is applied only once to decide to play hurt. Those who chose to rest despite high SC referred to either Maximin (50%) or Least probable negative (50%). Within the low SC group, the Maximin heuristic is mostly used when the choice to rest is made (86.7%). Still, 13.33% within this group found some RDOs to defuse the risk of SC induced by having a break. The heuristics referred to by the athletes are significantly different between the two conditions (Fisher’s exact test, *p* < 0.01). The effect is considerably large (Cramer’s *V* = 0.886).

## Discussion

Return to play is considered a key issue in the current sports medical discussion (Ardern et al., 2016). In order to better understand such decision-making processes, research including sociological and psychological approaches is necessary (Shrier et al., 2010). Moreover, there is the general challenge to develop and shape theories that are concerned with specific judgment and decision-making tasks in sport (Raab et al., 2019).

The main goal of our study was to analyze athletes’ decision-making strategies in return-to-play decisions using an experimental design. Following general Risky Decision Making Theory (Huber, 2012) with its focus on active risk defusing, we developed a theoretical model for understanding the process of return-to-play decision making from an athlete’s perspective. In accordance with this model (see Figure 1), a quasi-naturalistic return-to-play scenario was created to empirically assess amateur team sport athletes’ decision-making strategies under variation of social pressure to play hurt. To the best of our knowledge, our study is the first to use an experimental approach to analyze return-to-play decisions and the first to apply the scenario based method of active information search in the field of sports science.

### Information Acquisition and Return-to-Play Decision Making

A central goal of the study was to investigate which types of information team sport athletes need for return-to-play decision making. The identified information search patterns reveal different approaches to risky decision making: Not even one-third of all athletes follow a comprehensive information search in an attempt to get the full picture of the situation and its possible consequences. Thus, well-informed decisions covering the core areas of the framework of strategic assessment of risk and risk tolerance (Shrier et al., 2015) appear to be the exception rather than the rule. The most common search strategy is a highly focused search, with an interest in only sports or only medical related information. The athlete group which requested sports related infomation only, in particular, obviously shows no interest in potential medical consequences or medical background information about the condition. This selective information request can thus lead to highly biased decision making by neglecting medical risks. On the other hand, we see athletes who base their choice solely on medical information. This can be interpreted as a more cautious approach in terms of taking health risks.

Although justification pressure was applied, one-third of all athletes decided without actively searching for additional information at all. In other experiments where people were told to justify their decision afterward, an intensified search for information could be observed (Huber et al., 2009). This decreased information request in our study can be explained by the expert status of athletes. Experts usually know which kind of information is crucial for them to decide in risky situations (Huber et al., 2011a). Furthermore, background knowledge reduces the search intensity (Huber and Huber, 2008). As our supraspinatus scenario reflects a realistic injury, athletes probably have already developed mental representations of similar situations and therefore do not need additional background information for their decision. However, the observed tendency of lower information requests could also be interpreted as an indicator for routinized decision making based on already developed simple heuristics.

Within the group of athletes actively searching for information, details about potential sporting consequences of taking a break are crucial for most players. This indicates that the alternative to rest is most thoroughly evaluated. However, the results about initially requested information reveal that most athletes consider the medically risky alternative to play as the most promising one and stop searching to decide after SC-information is retrieved. Thus, the athletes evaluate, whether they can afford to rest by primarily considering potential social consequences. In particular, information about the relevance of the game or the availability of adequate substitutes is highly relevant for athletes in decision making. In contrast, medically relevant information about the characteristics and consequences of the health problem is only of subordinate importance. This general focus on consequences related information is in line with previous research on risky decision making using quasi-naturalistic scenarios (Huber, 2012). Our findings also support the hypothesis that people are not interested in probability information in real life situations (Tyszka and Zaleskiewicz, 2006). People obviously do not rely on probabilities, they rather orientate themselves on their own experience (Huber and Macho, 2001).

In contrast to the foregoing research on active information search, athletes only rarely requested RDO related information by active means. This might lead to the assumption that RDOs are irrelevant for the players. However, this is not the case, as the analysis of justification texts reveals. RDOs are mostly used in the written explanations of those athletes who chose the playing hurt alternative. Obviously, players refer to implicit anecdotal RDOs and do not need to actively search for this type of information. However, the lack of RDO search could also be explained by the design of our decision scenario. Confronting real-life athletes with a typical sports injury situation is way less hypothetical than the naturalistic decision scenarios used in basic risky decision research with inexperienced participants (e.g., Huber et al., 2011a). The identified RDOs in our study mostly refer to additional actions, which are typically used within sports to manage health problems, such as playing with reduced intensity, getting tape bandaged or pain medicated. Interestingly, we also identified RDOs for taking a (longer) break, such as having an adequate substitute available within the team. Thus, a sufficient number of alternate players in a team can be interpreted as a social resource that fosters health oriented decision making.

### Effects of High and Low Sporting Consequences (SC) on Return-to-Play Decision Making

The group confronted with high SC of having a break did not search more comprehensively for information than those who received low SC-information. This finding might be explained by the crucial role of this information category within the decision making process for both groups, as most search processes are terminated when high and low SC information is received. Perceiving higher social consequences also does not lead to an increased active information search for RDOs, which further underlines the fundamental role implicit RDOs must have in expert decision making.

Results show that RDOs play a major role when it comes down to making risky decisions in the sports context. If an acceptable RDO for medical consequences is identified, the chances of playing hurt are extremely high, particularly if high SC are perceived. As the subgroup analyses including the 33 athletes requesting SC-information reveal, decisions to play hurt almost always refer to the detection of an RDO. This means that if athletes detect severe SC, they subjectively minimize the risks of playing hurt by an additional action. The alternative to rest is mostly chosen based on Maximin-heuristics if the SC are high and no acceptable RDO can be found or when these are perceived as low. The use of Maximin-related justifications indicates that these athletes compared both alternatives to choose the one with subjectively less severe consequences.

These findings are in line with general risky decision making theory (Huber, 2012) and emphasize the central role of RDOs in expert decision making (Shiloh et al., 2006). Thus, our experiment confirms the finding of foregoing studies that the detection of an RDO is a very good predictor of choice. However, the analysis shows that RDOs become particularly relevant when the SC are high and consequently, social pressure to compete is increased. Due to the fact that an RDO gives the deciders some perceived controllability over the risk in question (Bär and Huber, 2008), the formerly risky decision subjectively becomes far less risky. Against the background of socialization processes into the culture of risk (Nixon, 1993), we assume that athletes learn from their sports networks a whole set of pre- and post-event RDOs. As the riskiness of an actually dangerous alternative is changed dramatically as soon as an RDO is incorporated into the mental representation, this learning process lays the foundation for playing hurt. However, this holds true only for situations with SC of having a break. In other situations, where social pressure to play is low, the athletes usually put health issues first.

Gender did not affect information search and decision making in our study. This observation is in line with recent research on the willingness to play hurt in elite sports (Mayer and Thiel, 2018; Mayer et al., 2018) and on managing pain and injury in elite sports (Young and White, 1995). The type of sport, in contrast, could be relevant due to an influence of sport discipline specific risk cultures (Mayer et al., 2018). However, the only difference we found was that basketball players were less interested in additional medical and sports related information acquisition than volleyball and handball players. It could be that a slight partial tear of the supraspinatus tendon is perceived as less severe in basketball than in handball and volleyball, where far more stress is applied on the injured structure when shooting. This might lead to different perception of the basic riskiness of the scenario. Thus, the assumption that the sports discipline influences risky decision making needs to be further analyzed.

### Limitations and Future Research

The findings about partly restrictive information search emphasize the potential restrictions of this method when applied in expert research. Although we ruled out that the participants had sustained an injury like the one we used in our scenario, it is still possible that knowledge from past injuries and/or shared experiences with teammates pre-structured the mental representations of the participants. Future studies including experts should therefore additionally include a totally unknown medical problem. This could also help to reduce the activation of potential medical background knowledge, to get a clearer picture of participants’ fundamental information needs.

Another limitation is rooted in the scenario method. It cannot be ruled out that athletes decide differently when they have supraspinatus injury in reality. However, this argument applies to every quasi-naturalistic study with an experimental design. Another potential limitation could result from the fact that the heuristics are based on written justification texts. The thinking aloud method (Bär and Huber, 2008) would perhaps give more insights into the information processing during the search process and therefore allow – combined with the justification texts – a more thorough reconstruction of the heuristics used for the decision.

For future research, it is necessary to include the perspectives of significant others, such as team doctors and coaches, as return-to-play is mostly the result of shared decision making. To further analyze the role of RDOs within return-to-play decision making, future studies should also develop and apply alternative risky decision making tasks, which include different kinds of both common and uncommon health problems. This methodical extension would allow to analyze the effects of activated background knowledge and established decision heuristics and implicit RDOs. The role of implicit RDOs in particular in return-to-play decision making should be analyzed more thoroughly in future research.

In order to improve health promotion strategies in competitive sports, future studies should analyze which social risks are associated with a decision against return-to-play while still injured. Here, it needs also to be evaluated, under which personal (e.g., one’s injury experiences) and social circumstances (e.g., leadership style of the coach or position within the team) specific RDOs are considered as acceptable. In longitudinal studies, it would be relevant to analyze whether risky decisions lead to long-term health problems and – in contrast – to what extent injury related “biographical disruptions” (Bury, 1982) are associated with more comprehensive and informed choices.

### Conclusion

A better understanding of return-to-play decision making helps to improve prevention and rehabilitation in sports, particularly when shared decision making is required (Dijkstra et al., 2017). Our results show that players can be easily persuaded to play despite injury if acceptable pre- and post-event RDOs are available. Thus, team doctors and coaches should be very careful in offering RDO-related information. For example, if a team apparently needs support of an injured player, mentioning the option “to start competing and see how the pain develops” is an effective way to steer the athlete’s decision toward “play.” The same holds true for risk defusing recommendations like “play at less than 100%” or “stop playing when the pain increases.” Offering RDOs like these trivialize health risks and promote playing hurt. In contrast, players can be easily convinced to rest if they are told that choosing the medically safe alternative does not have severe sporting consequences, like the loss of a starting position.

In general, doctors and coaches working with team sport athletes need to be aware that athletes are very sensitive to social expectations to play despite health issues. Doctors and coaches should also be aware that most athletes’ decisions are based on an incomprehensive information base. Only a few players favor very well-informed decisions based on a thorough risk analysis as suggested in the normative StARRT framework. Therefore, we strongly recommend that such normative return-to-play decision-making instruments need to incorporate the concept of RDOs as a major factor in everyday decision making.

## Data Availability Statement

The datasets generated for this study are available on request to the corresponding author.

## Ethics Statement

Ethical review and approval was not required for the study on human participants in accordance with the local legislation and institutional requirements. The patients/participants provided their written informed consent to participate in this study.

## Author Contributions

JM, SB, and AT designed the study and the experiments, analyzed and interpreted the data, and edited the manuscript and approved the final version to be published and are accountable for all aspects of the work. SB and JM performed the experiments. JM wrote the initial draft of the manuscript.

## Conflict of Interest

The authors declare that the research was conducted in the absence of any commercial or financial relationships that could be construed as a potential conflict of interest.
